# External validation of the Electronic Screening Index of Frailty (e-SIF) in a population of 1.4 million inhabitants aged 65 years and older

**DOI:** 10.1093/eurpub/ckag025

**Published:** 2026-02-16

**Authors:** Mateu Serra-Prat, Àngel Lavado, Emili Burdoy, Mònica Papiol, Laura Muñoz, Mateu Cabré

**Affiliations:** Research Unit, Consorci Sanitari del Maresme, Mataró, Barcelona, Spain; Networked Biomedical Research Centre for Liver and Digestive Diseases (CIBEREHD), Madrid, Spain; Research Unit, Germans Trias i Pujol Research Institute, Badalona, Barcelona, Spain; Information Management Unit, Consorci Sanitari del Maresme, Mataró, Barcelona, Spain; Primary Care Department, Consorci Sanitari del Maresme, Mataró, Barcelona, Spain; Primary Care Department, Consorci Sanitari del Maresme, Mataró, Barcelona, Spain; Datexbio SL, Barcelona, Spain; Research Unit, Consorci Sanitari del Maresme, Mataró, Barcelona, Spain

## Abstract

The Electronic Screening Index of Frailty (e-SIF) was first validated in 2022 in a sample of 9315 people, but further studies in larger populations were recommended to corroborate those findings. The objective was to evaluate e-SIF construct validity, in a large and independent sample from that used for its initial validation, by analysing score associations with age, sex, hospitalizations, institutionalizations, mortality, and health resource use. An observational 2-year longitudinal study (2018–2019) was conducted of the Catalan population aged ≥65 years (1.4 million people) using retrospectively collected data. Frailty was established according to e-SIF score. Study variables included sociodemographic characteristics, mortality, hospitalizations, institutionalizations, primary care visits, emergency visits, and day hospital sessions during the study period. The study included 1 465 312 people (mean age 75.8 years, 57.2% women). Frailty prevalence was 14.0% in women and 9.1% in men (*P* < .001) and progressively increased with age from 2.2% in the 65–69 age group to 38.9% in ≥95 age group. As frailty status increased, mortality, hospitalizations, institutionalizations, and health resource use also increased. Hospitalization-free survival and institutionalization-free survival worsened as frailty increased. Multivariate logistic regression models based on all 42 e-SIF items showed areas under the curve of 0.85, 0.75, and 0.82 in predicting 1-year mortality, hospitalizations, and institutionalizations, respectively. This large study corroborates the findings of the initial e-SIF validation study and reaffirms its good construct validity.

Key points

- The e-SIF behaves as expected in relation to age and sex and accurately predicts mortality, hospitalizations, and institutionalizations.
- The e-SIF automatically classifies aged population into the following categories: robust, pre-frail, moderately frail, and severely frail.
- The e-SIF is a valid instrument for classifying frailty status in the community and for frailty screening.

## Introduction

Frailty, a prevalent clinical condition in the aged population [1], has a high impact on quality of life [2] and on health and social resource use and costs [3]. Frailty is reversible, especially in its initial phases [4]. Multicomponent interventions include good control of underlying chronic diseases, rational use of medication, measures to prevent falls, physical exercise, good dietary habits, emotional wellbeing, and social issues [5, 6]. The World Health Organization (WHO) and other international scientific bodies recommend frailty screening of the aged population to prevent functional decline, disability, and dependency [7–9]. In recent years, interest has grown in automated frailty screening of the aged population, as traditional manual instruments are time-consuming to administer and so pose an excessive burden for healthcare professionals, especially in primary care [10].

The Electronic Screening Index of Frailty (e-SIF) automatically identifies frailty status in the population aged ≥65 years, at any given moment in time. It does so using data routinely recorded in Health Information System databases, specifically, 42 clinical conditions (related to diseases, medication, geriatric syndromes, symptoms, risk factors, biomarkers, and social characteristics), along with their start and end dates. Each clinical condition is defined by a group from the International Classification of Diseases, Tenth Revision (ICD-10), Anatomical Therapeutic Chemical (ATC) codes, or an algorithm combining codes and other variables. Summed scores for the clinical conditions yield the following four frailty categories: robust (0–4 points), pre-frail (5–8 points), moderately frail (9–11 points), and severely frail or dependent (≥12 points). An initial validation in a sample of 9315 people in the Maresme region of Catalonia (Spain) in 2022, revealed, as expected, that the e-SIF was associated with age, sex, hospitalizations, institutionalizations (in a social healthcare centre), health resource use, and mortality. While e-SIF validity was satisfactory, further study of a larger population was necessary to corroborate these findings [11].

The aim of this study was to evaluate e-SIF construct validity in a large sample, independent from that used for its initial validation, and to analyse score associations with age, sex, mortality, hospitalizations, institutionalizations, and health resource use.

## Methods

### Study design and population

Using retrospectively collected data, we conducted an observational 2-year longitudinal study (31 December 2017–31 December 2019) of the Catalan population aged ≥65 years (*n* = 1 415 643 in 2017). Catalonia, an autonomous region in northeast Spain with 7.5 million inhabitants in 2017, has a universal public healthcare system with 98% healthcare coverage. The study protocol was approved by the local ethics committee (reference CEIm CSdM 09/22).

### Data sources

A central registry of insured persons in Catalonia links primary care, hospital, pharmacy, and laboratory data, coded according to the ICD-10 and ATC classification systems. The Catalan Department of Health periodically implements a validation process to check data consistency and to identify potential errors, and it also conducts external audits to ensure data quality and reliability. An agreement was signed with the Agency for Health Quality and Assessment of Catalonia (AQUAS) regarding justified access to pre-established pseudo-anonymized health data for research purposes, as permitted under the Public Data Analysis for Health Research and Innovation (PADRIS) 2022 programme. PADRIS data engineers accordingly designed processes for automatically extracting the required data, to which quality control, data cleaning, and pseudo-anonymization techniques were applied. Extracted data were stored on an AQUAS server, to which the researchers were given access for the analysis. Only aggregated data could be retrieved, always authorized by and under the supervision of PADRIS technicians.

### Study variables

Data needed to calculate e-SIF frailty status were collected as follows: age, sex, health conditions (ICD-10 codes), dispensed medication (ATC codes), and other clinical characteristics (body mass index, blood count, basic biochemistry, and Barthel score). e-SIF scores and frailty status were calculated for 31 December 2017 according to criteria, codes, and algorithms described elsewhere [11]. Data were also collected during the study period on mortality, hospitalizations, institutionalizations, primary care visits, emergency visits, and day hospital sessions, along with the corresponding dates.

### Statistical analysis

e-SIF scores were described using means, standard deviation (SD), medians, and interquartile range (IQR) for the entire study population and by sex and age group (65–69, 70–74, 75–79, 80–84, 85–89, 90–94, and ≥95 years). The Mann–Whitney *U* test and the Kruskal–Wallis test were used to compare e-SIF scores by sex and age groups, respectively. Prevalence of both frailty (moderate + severe frailty; e-SIF ≥ 9) and severe frailty (e-SIF ≥ 12) were calculated for the entire study population and by sex and age group. The chi-square test was used to compare proportions between groups. A linear trend test in one-way analysis of variance (ANOVA) was used to compare e-SIF scores by age group, and mortality, hospitalization and institutionalization rates by frailty category. The relationship between frailty category and health resource use was determined by comparing the mean number of primary care, emergency, and day hospital visits for the different frailty categories using ANOVA. Multivariate logistic regression models to predict 1- and 2-year mortality, non-planned hospitalizations, and institutionalizations included age, sex, and all 42 e-SIF clinical conditions as independent variables. The models were tested using a randomly selected 70% of the study population (*n* = 990 950), while the remaining 30% of the study population (*n* = 424 693) was used to assess model validity in predicting the outcome measures, based on calculating the area under the receiver operating characteristic curve (AUC). In addition, 1- and 2-year mortality, hospitalization, and institutionalization risks for people aged ≥65 years were calculated as follows:


$$\text{probability}=1/\left(1+e^{-z}\right),$$

where *e* is the natural logarithm, and where *z* is *b*_0_+*b*_1_·x_1_+*b*_2_·x_2_+…+*b_n_*·*x_n_*, with *b*_0_ as the intercept, *b*_1_, *b*_2_, … *b_n_* as the regression coefficients, and *x*_1_, *x*_2_ … *x_n_* as the values of the independent or predictor variables (age, sex, and the 42 e-SIF items). Finally, hospital-free survival and institutionalization-free survival for each frailty category were calculated using the Kaplan–Meier method, and survival curves were compared using the log rank test and bivariate and multivariate Cox regression (adjusted for age and sex). Note that overall survival could not be analysed, as access to date of death was not authorized. Statistical significance in all statistical tests was set to *P* < .05.

## Results

### Study population

Of a Catalan population of 7 496 276 inhabitants at the end of 2017, 18.9% (*n* = 1 465 312) were aged ≥65 years: mean age 75.8 (SD 7.9) years and 57.2% women. The main comorbidities were arterial hypertension (57.8%), arthritis (40.3%), diabetes (22.1%), chronic lung diseases (16.9%), sleep disorders (14.4%), kidney failure (13.6%), osteoporosis (13.4%), depression (12.5%), visual alterations (11.7%), and dyspepsia or gastroesophageal reflux (11.0%); furthermore, 28.7% were obese, 15.5% experienced urinary or faecal incontinence, and 53.9% were taking ≥5 medications.

### e-SIF score and frailty status by sex and age group

Table 1 summarizes e-SIF scores and prevalence of frailty categories by sex and age group in 2017. Mean e-SIF scores were 4.76 (SD 3.38) for women and 4.14 (SD 3.08) for men. The scores progressively increased with age from 3.02 (women) and 2.5 (men) for the 65–69 age group to 7.96 (women) and 3.92 (men) for the ≥95 age group. Frailty prevalence was 14.0% in women and 9.1% in men (*P* < .001) and progressively increased with age from 2.2% for the 65–69 age group to 38.9% for the ≥95 age group. Severe frailty (e-SIF score ≥12) prevalence was 3.9% in women and 2.1% in men (*P* < .001), and progressively increased with age from 0.28% for the 65–69 age group to 12.37% for the ≥95 age group.

**Table 1.** e-SIF scores and prevalence of frailty categories by sex and age in 2017

	e-SIF score				Prevalence of frailty categories				
	*N*	Mean (SD)	Median (IQR)	*P* or *P* for trend^a^	Robust (%)	Pre-frail (%)	Moderately Frail (%)	Severely frail (%)	*P*
All (year)	1 415 643	4.50 (3.27)	4 (2–6)	–	770 643 (54.4)	476 034 (33.6)	124 229 (8.8)	44 737 (3.2)	–
Women	809 395	4.76 (3.38)	4 (2–7)	<.001	414 743 (51.2)	280 860 (34.7)	82 046 (10.1)	31 746 (3.9)	<.001
Men	606 248	4.14 (3.08)	4 (2–6)		355 900 (58.7)	195 174 (32.2)	42 183 (7.0)	12 991 (2.1)	
65–69	383 341	3.02 (2.50)	3 (1–5)	<.001^a^	291 126 (75.9)	83 799 (21.9)	7345 (1.9)	1071 (0.3)	<.001
70–74	337 382	3.77 (2.76)	4 (2–5)		221 623 (65.7)	101 220 (30.0)	12 320 (3.7)	2219 (0.5)	
75–79	235 545	4.58 (3.07)	4 (2–6)		128 084 (54.7)	88 075 (37.4)	15 607 (6.6)	3779 (1.6)	
80–84	223 860	6.55 (3.43)	6 (4–9)		70 509 (31.5)	104 488 (46.7)	36 002 (16.1%)	12 861 (5.7%)	
85–89	150 904	7.32 (3.70)	7 (5–10)		39 475 (26.2)	65 289 (43.3)	31 759 (21.0%)	14 381 (9.5%)	
90–94	66 324	7.85 (3.85)	8 (5–11)		15 475 (23.3)	26 334 (39.7)	16 351 (24.7%)	8164 (12.3%)	
>95	18 287	7.96 (3.92)	8 (5–11)		4351 (23.8)	6829 (37.3)	4845 (26.5%)	2262 (12.4%)	

Abbreviations: IQR: interquartile range; SD: standard deviation.

a Indicates test for trend.

### Two-year mortality and health resource use by frailty category

Table 2 reports 2-year comparisons of mortality, hospitalizations, institutionalizations, primary care visits, emergency visits, day hospital sessions, and dispensed medication by frailty category; 1-year comparisons are reported in the Supplementary Appendix S1. Both tables show a consistent and significant trend of increased mortality and health resource use as frailty status increased. Comparing, for instance, the robust category to the severely frail category at 1 year, mortality increased from 1.8% to 21.3%, urgent hospitalizations increased from 5.0% to 30.4%, and institutionalizations increased from 1.3% to 15.2%.

**Table 2.** Two-year mortality and health resource use by frailty category (2018–2019)

	Robust	Pre-frail	Moderately frail	Severely frail	*P* for trend
**Mortality, *n* (%)**	28 695 (3.7%)	38 233 (8.0%)	26 847 (21.6%)	16 350 (36.5%)	<.001
**Hospitalizations, *n* (%)**	99 929 (13.0%)	115 516 (24.3%)	47 343 (38.1%)	21 154 (47.3%)	<.001
Urgent	71 067 (9.2%)	89 791 (18.9%)	42 007 (33.8%)	19 748 (44.1%)	<.001
Scheduled	42 639 (5.5%)	42 650 (9.0%)	12 188 (9.8%)	4447 (9.9%)	<.001
Mean (SD) hospitalizations	0.20 (0.6)	0.39 (0.9)	0.69 (1.2)	0.95 (1.5)	<.001
Urgent	0.13 (0.5)	0.28 (0.7)	0.56 (1.0)	0.81 (1.3)	<.001
Scheduled	0.07 (0.3)	0.11 (0.4)	0.13 (0.5)	0.14 (0.5)	<.001
**Institutionalizations, *n* (%)**	20 339 (2.6%)	33 720 (7.1%)	20 136 (16.2%)	10 655 (23.8%)	<.001
Mean (SD) institutionalizations	0.04 (0.2)	0.10 (0.4)	0.24 (0.7)	0.37 (0.8)	<.001
**Primary care visits, *n* (%)**	628 606 (81.6%)	467 905 (98.3%)	121 832 (98.1%)	43 723 (97.7%)	<.001
Mean (SD) primary care visits	17.2 (18.1)	36.1 (28.0)	48.9 (39.1)	54.2 (48.5)	<.001
**Emergency visits, *n* (%)**	342 354 (44.4%)	306 091 (64.3%)	96 329 (77.5%)	37 008 (82.7%)	<.001
Mean (SD) emergency visits	1.37 (2.7)	2.65 (4.1)	4.43 (5.8)	5.66 (7.2)	<.001
**Day hospital sessions, *n* (%)**	17 286 (2.2%)	20 432 (4.3%)	6711 (5.4%)	2709 (6.1%)	<.001
Mean (SD) day hospital sessions	0.09 (1.2)	0.17 (1.62)	0.22 (1.8)	0.28 (2.0)	<.001
**Dispensed medications, *n* (%)**	483 643 (62.8%)	424 905 (89.3%)	113 621 (91.5%)	41 539 (92.9%)	<.001
Mean (SD) dispensed medications (€)	416 (900)	1424 (1698)	2279 (2193)	2792 (2475)	<.001

Abbreviation: SD: standard deviation.

### Hospitalization- and institutionalization-free survival

**Figure 1.**
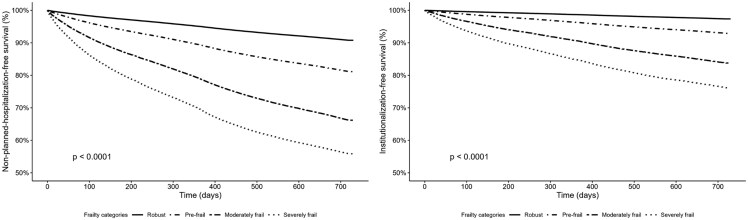
Hospitalization-free survival and institutionalization-free survival by frailty categories.

Figure 1, which compares hospitalization-free survival and institutionalization-free survival curves for the four frailty categories, shows that survival worsened in all cases as frailty increased.

### Predictive value of the multivariate logistic regression models

**Figure 2.**
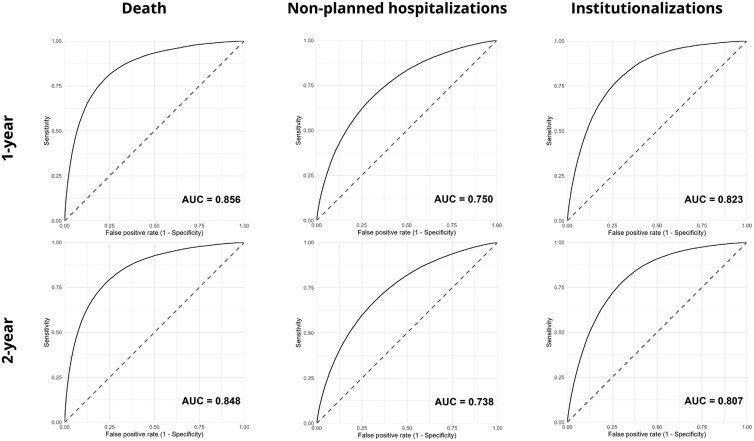
AUC of the e-SIF predictive models for 1- and 2-year mortality, hospitalization, and institutionalization in a social-healthcare centre.

AUC values in predicting 1- and 2-year outcomes, respectively, were as follows: mortality, 0.855 and 0.847; hospitalization, 0.750 and 0.737; and institutionalization, 0.823 and 0.806 (see Fig. 2). The regression coefficients, reported in the Supplementary Appendix S2, enable the creation of a calculator to estimate individual 1- and 2-year risks of mortality, hospitalization, and institutionalization.

## Discussion

This external validation study—in a large population sample different from that of the initial validation [11]—confirms that the e-SIF behaves as expected in relation to sex and age groups, in that it accurately predicts mortality, hospitalizations, and institutionalizations, and is positively correlated with health resource use (emergency and primary care visits, and day hospital sessions). Our results therefore reaffirm the validity of the e-SIF as a screening tool for frailty in the population aged ≥65 years.

We observed frailty prevalence of 13.0% (8.8% moderate and 3.2% severe) and, as expected, prevalence was both higher in women than in men and progressively increased with age, from 2.2% in the 65–69 age group to 38.9% in ≥90 age group. These results not only agree with those published regarding the first e-SIF validation [11], but they also agree with other studies of frailty prevalence measured with other instruments and in other settings and populations [1, 10]. The increase in frailty prevalence with age is in line with characterization of this clinical condition in terms of functional decline, a decrease in the body’s reserves, and a reduced ability to respond to and resist stressors [2]. This increase in frailty prevalence with age is the first evidence of e-SIF validity. Other evidence also points in this same direction, namely, the increase in mortality, hospitalizations, and institutionalizations with increased frailty.

Mortality clearly trends upwards as frailty severity increases, going from 3.6% for robust individuals to 36.5% for severely frail individuals, and corroborating the results reported for the first e-SIF validation process [11] and by other studies that used mortality to validate other frailty tools [10]. There is abundant evidence, in fact, that frailty is related to an increased risk of both all-cause mortality and cause-specific mortality from cardiovascular diseases, cancer, and respiratory illness in community-dwelling adults [12, 13]. The increase in mortality with frailty severity also agrees with the widely accepted notion that greater frailty or vulnerability leads to greater mortality due to the body’s mitigated capacity to react to stressors [3].

Likewise, in the same way and for the same reasons, hospitalizations, emergency visits, primary care visits, and day hospital sessions use, as expected, increased as frailty severity increased. Comparing, for instance, the robust and severely frail groups, we find that non-planned hospitalizations increased from 9.2% to 44.1%, mean emergency visits from 2.7 to 7.2, and mean primary care visits from 18.1 to 48.5. Moreover, dispensed medication cost went from €416 to €2792 in the robust and severely frail groups, indicating an approximately six-fold increase in drug expenditure for frail compared to robust individuals. It is worth noting that few studies have assessed the validity of frailty instruments for pharmaceutical expenditure comparisons according to frailty severity, despite evidence indicating that frailty is responsible for approximately a two and a half- to three-fold increase in healthcare costs [3, 14–16]. Regarding institutionalizations in social healthcare centres, this also increased with frailty, going from 2.6% in the robust group to 23.8% in the severely frail group. This was as expected, as greater frailty leads to a greater need for help with activities of daily living, especially during recovery from acute clinical situations [17].

Frailty screening programmes in the population aged ≥65 years require validated automatic instruments to classify frailty status. The implementation of such instruments and the automatic and updated loading of their results in primary care and/or hospital electronic clinical notes is not always easy and require the involvement of information management units to identify data sources and to design and implement automatic data extraction, transformation, and uploading processes. Healthcare institutions, however, frequently fail to prioritize the allocation of time and resources to the implementation of electronic frailty screening. Screening implementation remains a challenge [20], given the diversity of tools [18], the lack of awareness of the magnitude and consequences of frailty [19], and the workload of information management units, among other reasons.

Another way to implement e-SIF in clinical practice is by creating a computer-based calculator or using a web-based calculator, such as the QFrailty (https://qfrailty.org/) and QMortality (https://qmortality.org/) calculators [21]. These instantly calculate individual risk from manually input data, and automatically predict other risks, such as hospitalization or institutionalization. This kind of information would be a useful aid for healthcare providers in medical consultations. The logistic regression models based on the 42 e-SIF items showed high predictive validity for mortality, hospitalization, and institutionalization (with AUCs of 0.85, 0.75, and 0.82, respectively). These results are very similar to those reported for the QMortality and QFrailty calculators [21]. While the latter calculators are specifically designed for use in the UK (collected data include region, postal code, and ethnicity), the e-SIF, with very similar diagnostic validity and risk prediction accuracy, can be applied worldwide.

In summary, the e-SIF, as an automatic frailty screening tool for the aged population, has several strengths. First, construct validity has been evaluated in a very large population (1.4 million people), showing results very similar to those of the initial validation process. This external validation reaffirms the e-SIF’s good construct validity. Second, the e-SIF shows very good predictive and psychometric properties for 1- and 2-year mortality, hospitalizations, and institutionalizations, all of interest in clinical practice. Third, the fact that the e-SIF uses ICD-10 codes and that all item definitions and algorithms are publicly available facilitates international use and comparison. Fourth, like other similar electronic tools, the e-SIF enables large-scale, automatic, and instantaneous frailty assessments that can be included in computerized medical records. Finally, also available—possibly facilitating its use in medical consultations—is a manual version of the e-SIF (SIF calculator) that also calculates 1- and 2-year mortality, hospitalization, and institutionalization risks.

The e-SIF also has certain limitations. Because it uses routinely recorded data, output quality depends on the quality of records and coding in electronic clinical records and healthcare databases. Furthermore, as a screening tool, it cannot replace a comprehensive geriatric assessment aimed at confirming a frailty diagnosis in clinical practice. Finally, the lack of a gold standard or a universally accepted operational definition of frailty means that criterion validity cannot be assessed, i.e. how e-SIF results compare with a reference frailty criterion. For those reasons, further studies should corroborate e-SIF validity in different health systems and in comparisons with other validated and widely used frailty instruments.

In conclusion, for a new and large population sample of close to 1.5 million persons aged ≥65 years, our study corroborates the findings of the initial e-SIF validation study, reinforcing its good construct validity. The e-SIF, which behaves as expected in relation to age and sex, classifies individuals in one of four categories according to frailty status (robust, pre-frail, moderately frail, and severely frail), each with clearly differentiated and progressively increased risks of death, hospitalization, and institutionalization, and with progressively increased social and health resource use. These findings strongly suggest that the e-SIF is a valid instrument for classifying frailty status in the community.

## Supplementary Material

ckag025_Supplementary_Data

## Data Availability

The dataset from this study is held securely in an AQUAS server. Access must be requested from AQUAS at padris@gencat.cat. The AQUAS website (https://aquas.gencat.cat) explains who can access the dataset and the conditions for access.
