# Supplementary material for: External validation of the Electronic Screening Index of Frailty (e-SIF) in a population of 1.4 million inhabitants aged 65 years and older
Source: Eur J Public Health. 2026 Feb 16;36(2):ckag025. doi: 10.1093/eurpub/ckag025 (PMC13168815; doi:10.1093/eurpub/ckag025)
Supplement: ckag025_Supplementary_Data [file ckag025_supplementary_data.zip › ejph-2025-11-om-0986-File007.docx]

**Supplementary Material. Appendix 2.** One-year (2018) and two-year (2018-2019) death, urgent hospitalization, and institutionalization probabilities for persons with specific characteristics.

**A. Probability of death**

y=death at one or two years (regression model fitted for each outcome)

x=adjustment variables=sex+age+41 e-SIF items; item 39 (age >80 years) is excluded as it correlates with age

**Model coefficients**

|  | **2018 (at 1 year)** | | | **2019 (at 2 years)** | | |
| --- | --- | --- | --- | --- | --- | --- |
|  | **Coefficient (β)** | **95% CI** | **p** | **Coefficient (β)** | **95% CI** | **p** |
| *Intercept* | -11.590 |  |  | -11.512 |  |  |
| **Sex** |  |  |  |  |  |  |
| Women | Ref. | - | - | Ref. | - | - |
| Men | 0.436 | (0.411; 0.461) | <0.001 | 0.481 | (0.462; 0.500) | <0.001 |
| **Age (y)** | 0.098 | (0.096; 0.100) | <0.001 | 0.106 | (0.105; 0.108) | <0.001 |
| **e-SIF items** |  |  |  |  |  |  |
| Item1 | -0.070 | (-0.143; 0.004) | 0.063 | -0.117 | (-0.176; -0.057) | <0.001 |
| Item2 | 0.319 | (0.284; 0.354) | <0.001 | 0.349 | (0.320; 0.377) | <0.001 |
| Item3 | -0.298 | (-0.321; -0.274) | <0.001 | -0.284 | (-0.302; -0.266) | <0.001 |
| Item4 | 0.203 | (0.172; 0.233) | <0.001 | 0.246 | (0.222; 0.270) | <0.001 |
| Item5 | -0.068 | (-0.117; -0.018) | 0.007 | -0.043 | (-0.082; -0.004) | 0.030 |
| Item6 | 0.041 | (0.013; 0.068) | 0.004 | 0.081 | (0.059; 0.102) | <0.001 |
| Item7 | 0.152 | (0.126; 0.178) | <0.001 | 0.183 | (0.163; 0.203) | <0.001 |
| Item8 | -0.094 | (-0.150; -0.038) | 0.001 | -0.102 | (-0.145; -0.058) | <0.001 |
| Item9 | 0.202 | (0.163; 0.241) | <0.001 | 0.206 | (0.175; 0.237) | 0 |
| Item10 | 0.037 | (-0.062; 0.137) | 0.464 | 0.035 | (-0.046; 0.116) | 0.397 |
| Item11 | -0.227 | (-0.262; -0.193) | <0.001 | -0.225 | (-0.251; -0.199) | <0.001 |
| Item12 | 0.433 | (0.398; 0.468) | <0.001 | 0.511 | (0.483; 0.539) | <0.001 |
| Item13 | 0.543 | (0.493; 0.593) | <0.001 | 0.587 | (0.546; 0.627) | <0.001 |
| Item14 | -0.246 | (-0.272; -0.221) | <0.001 | -0.248 | (-0.267; -0.229) | <0.001 |
| Item15 | -0.236 | (-0.286; -0.186) | <0.001 | -0.254 | (-0.292; -0.216) | <0.001 |
| Item16 | 0.033 | (-0.009; 0.076) | 0.122 | 0.047 | (0.014; 0.080) | 0.005 |
| Item17 | 0.418 | (0.385; 0.451) | <0.001 | 0.523 | (0.497; 0.549) | <0.001 |
| Item18 | -0.143 | (-0.177; -0.110) | <0.001 | -0.137 | (-0.163; -0.112) | <0.001 |
| Item19 | 0.296 | (0.237; 0.355) | <0.001 | 0.402 | (0.355; 0.449) | <0.001 |
| Item20 | 0.587 | (0.558; 0.616) | <0.001 | 0.626 | (0.603; 0.649) | <0.001 |
| Item21 | -0.166 | (-0.204; -0.129) | <0.001 | -0.165 | (-0.193; -0.137) | <0.001 |
| Item22 | 0.269 | (0.226; 0.313) | <0.001 | 0.301 | (0.268; 0.335) | <0.001 |
| Item23 | -0.084 | (-0.110; -0.059) | <0.001 | -0.071 | (-0.091; -0.052) | <0.001 |
| Item24 | 0.100 | (0.072; 0.128) | <0.001 | 0.128 | (0.107; 0.149) | <0.001 |
| Item25 | 0.609 | (0.561; 0.657) | <0.001 | 0.614 | (0.572; 0.656) | <0.001 |
| Item26 | -0.016 | (-0.045; 0.014) | 0.298 | -0.044 | (-0.067; -0.021) | <0.001 |
| Item27 | -0.019 | (-0.060; 0.022) | 0.372 | -0.021 | (-0.052; 0.010) | 0.177 |
| Item28 | 0.424 | (0.396; 0.453) | <0.001 | 0.419 | (0.397; 0.441) | <0.001 |
| Item29 | 0.208 | (0.150; 0.265) | <0.001 | 0.208 | (0.161; 0.255) | <0.001 |
| Item30 | -0.147 | (-0.291; -0.004) | 0.044 | -0.145 | (-0.253; -0.036) | 0.009 |
| Item31 | 0.451 | (0.383; 0.519) | <0.001 | 0.457 | (0.405; 0.510) | <0.001 |
| Item32 | -0.208 | (-0.235; -0.180) | <0.001 | -0.191 | (-0.211; -0.170) | <0.001 |
| Item33 | -0.041 | (-0.125; 0.042) | 0.331 | -0.015 | (-0.081; 0.051) | 0.663 |
| Item34 | -0.020 | (-0.053; 0.012) | 0.212 | -0.002 | (-0.026; 0.023) | 0.904 |
| Item35 | -0.086 | (-0.146; -0.026) | 0.005 | -0.139 | (-0.188; -0.090) | <0.001 |
| Item36 | 0.600 | (0.573; 0.627) | <0.001 | 0.561 | (0.540; 0.582) | <0.001 |
| Item37 | 0.211 | (0.113; 0.311) | <0.001 | 0.203 | (0.124; 0.283) | <0.001 |
| Item38 | 0.400 | (-0.102; 0.902) | 0.118 | 0.509 | (0.143; 0.876) | 0.006 |
| Item39 | - | - | - | - | - | - |
| Item40 | 0.294 | (0.214; 0.374) | <0.001 | 0.255 | (0.185; 0.326) | <0.001 |
| Item41 | 1.092 | (1.066; 1.118) | <0.001 | 0.980 | (0.959; 1.001) | <0.001 |
| Item42 | 0.395 | (0.321; 0.468) | <0.001 | 0.459 | (0.404; 0.514) | <0.001 |

**B. Probability of urgent hospitalization**

y=urgent hospitalization at one or two years (regression model fitted for each outcome)

x=adjustment variables=sex+age+41 e-SIF items; item 39 (age >80 years) is excluded as it correlates with age

**Model coefficients**

|  | **2018 (at 1 year)** | | | **2019 (at 2 years)** | | |
| --- | --- | --- | --- | --- | --- | --- |
|  | **Coefficient (β)** | **95% CI** | **p** | **Coefficient (β)** | **95% CI** | **p** |
| *Intercept* | -6.665 |  |  | -6.313 |  |  |
| **Sex** |  |  |  |  |  |  |
| Women | Ref. | - | - | Ref. | - | - |
| Men | 0.322 | (0.306; 0.339) | <0.001 | 0.335 | (0.322; 0.349) | <0.001 |
| **Age (in years)** | 0.046 | (0.045; 0.047) | <0.001 | 0.050 | (0.049; 0.051) | <0.001 |
| **e-SIF item** |  |  |  |  |  |  |
| Item1 | 0.116 | (0.062; 0.171) | <0.001 | 0.161 | (0.115; 0.208) | <0.001 |
| Item2 | 0.264 | (0.238; 0.291) | <0.001 | 0.285 | (0.262; 0.308) | <0.001 |
| Item3 | -0.070 | (-0.085; -0.055) | <0.001 | -0.051 | (-0.063; -0.038) | <0.001 |
| Item4 | 0.316 | (0.295; 0.338) | <0.001 | 0.344 | (0.326; 0.362) | <0.001 |
| Item5 | 0.116 | (0.082; 0.150) | <0.001 | 0.147 | (0.119; 0.176) | <0.001 |
| Item6 | 0.183 | (0.164; 0.201) | <0.001 | 0.202 | (0.186; 0.217) | <0.001 |
| Item7 | 0.228 | (0.211; 0.245) | <0.001 | 0.244 | (0.231; 0.258) | <0.001 |
| Item8 | 0.036 | (-0.002; 0.073) | 0.062 | 0.053 | (0.023; 0.084) | 0.001 |
| Item9 | 0.246 | (0.220; 0.272) | <0.001 | 0.243 | (0.221; 0.265) | <0.001 |
| Item10 | 0.111 | (0.035; 0.187) | 0.004 | 0.155 | (0.091; 0.220) | <0.001 |
| Item11 | 0.043 | (0.022; 0.064) | <0.001 | 0.072 | (0.055; 0.089) | <0.001 |
| Item12 | 0.411 | (0.386; 0.436) | <0.001 | 0.425 | (0.403; 0.447) | <0.001 |
| Item13 | -0.198 | (-0.239; -0.158) | <0.001 | -0.320 | (-0.354; -0.285) | <0.001 |
| Item14 | -0.086 | (-0.103; -0.069) | <0.001 | -0.066 | (-0.079; -0.052) | <0.001 |
| Item15 | -0.005 | (-0.036; 0.025) | 0.730 | -0.008 | (-0.032; 0.017) | 0.551 |
| Item16 | 0.173 | (0.145; 0.201) | <0.001 | 0.199 | (0.176; 0.222) | <0.001 |
| Item17 | 0.051 | (0.023; 0.079) | <0.001 | 0.061 | (0.037; 0.084) | <0.001 |
| Item18 | 0.013 | (-0.009; 0.036) | 0.232 | 0.013 | (-0.004; 0.031) | 0.140 |
| Item19 | 0.175 | (0.128; 0.222) | <0.001 | 0.194 | (0.155; 0.234) | <0.001 |
| Item20 | 0.237 | (0.214; 0.260) | <0.001 | 0.205 | (0.185; 0.225) | <0.001 |
| Item21 | -0.013 | (-0.035; 0.010) | 0.268 | -0.019 | (-0.037; -0.001) | 0.039 |
| Item22 | 0.308 | (0.279; 0.337) | <0.001 | 0.328 | (0.303; 0.352) | <0.001 |
| Item23 | 0.117 | (0.100; 0.134) | <0.001 | 0.125 | (0.112; 0.139) | <0.001 |
| Item24 | 0.373 | (0.356; 0.390) | <0.001 | 0.368 | (0.353; 0.382) | <0.001 |
| Item25 | 0.150 | (0.106; 0.195) | <0.001 | 0.112 | (0.073; 0.152) | <0.001 |
| Item26 | -0.033 | (-0.052; -0.013) | 0.001 | -0.038 | (-0.054; -0.022) | <0.001 |
| Item27 | 0.130 | (0.103; 0.157) | <0.001 | 0.164 | (0.142; 0.186) | <0.001 |
| Item28 | 0.194 | (0.174; 0.214) | <0.001 | 0.175 | (0.159; 0.192) | <0.001 |
| Item29 | 0.166 | (0.123; 0.210) | <0.001 | 0.209 | (0.173; 0.246) | <0.001 |
| Item30 | 0.142 | (0.059; 0.224) | 0.001 | 0.159 | (0.092; 0.226) | <0.001 |
| Item31 | 0.309 | (0.265; 0.353) | <0.001 | 0.284 | (0.247; 0.321) | <0.001 |
| Item32 | 0.050 | (0.034; 0.067) | <0.001 | 0.066 | (0.053; 0.080) | <0.001 |
| Item33 | 0.121 | (0.063; 0.180) | <0.001 | 0.107 | (0.058; 0.156) | <0.001 |
| Item34 | 0.077 | (0.056; 0.099) | <0.001 | 0.085 | (0.068; 0.102) | <0.001 |
| Item35 | -0.160 | (-0.206; -0.113) | <0.001 | -0.155 | (-0.196; -0.115) | <0.001 |
| Item36 | 0.262 | (0.243; 0.281) | <0.001 | 0.240 | (0.224; 0.256) | <0.001 |
| Item37 | 0.239 | (0.166; 0.312) | <0.001 | 0.271 | (0.210; 0.331) | <0.001 |
| Item38 | 0.432 | (0.142; 0.722) | 0.004 | 0.523 | (0.290; 0.755) | <0.001 |
| Item39 | - | - | - | - | - | - |
| Item40 | 0.045 | (-0.023; 0.114) | 0.191 | 0.028 | (-0.032; 0.087) | 0.357 |
| Item41 | 1.044 | (1.025; 1.062) | <0.001 | 0.942 | (0.926; 0.959) | <0.001 |
| Item42 | 0.399 | (0.355; 0.443) | <0.001 | 0.449 | (0.412; 0.485) | <0.001 |

**C. Probability of institutionalization**

y=institutionalization at one or two years (regression model fitted for each outcome)

x=adjustment variables=sex+age+41 e-SIF items; item 39 (age >80 years) is excluded as it correlates with age

**Model coefficients**

|  | **2018 (at 1 year)** | | | **2019 (at 2 years)** | | |
| --- | --- | --- | --- | --- | --- | --- |
|  | **Coefficient (β)** | **95% CI** | **p** | **Coefficient (β)** | **95% CI** | **p** |
| *Intercept* | -10.375 |  |  | -9.943 |  |  |
| **Sex** |  |  |  |  |  |  |
| Women | - | - | - | - | - | - |
| Men | 0.102 | (0.075; 0.129) | <0.001 | 0.109 | (0.088; 0.129) | <0.001 |
| **Age (in years)** | 0.079 | (0.078; 0.081) | <0.001 | 0.083 | (0.081; 0.084) | <0.001 |
| **e-SIF item** |  |  |  |  |  |  |
| Item1 | 0.262 | (0.190; 0.334) | <0.001 | 0.260 | (0.202; 0.319) | <0.001 |
| Item2 | 0.175 | (0.137; 0.213) | <0.001 | 0.196 | (0.166; 0.226) | <0.001 |
| Item3 | 0.008 | (-0.017; 0.032) | 0.542 | 0.010 | (-0.009; 0.028) | 0.314 |
| Item4 | 0.185 | (0.153; 0.218) | <0.001 | 0.229 | (0.204; 0.254) | <0.001 |
| Item5 | 0.182 | (0.133; 0.231) | <0.001 | 0.193 | (0.154; 0.232) | <0.001 |
| Item6 | 0.033 | (0.004; 0.062) | 0.025 | 0.072 | (0.050; 0.094) | <0.001 |
| Item7 | 0.201 | (0.174; 0.228) | <0.001 | 0.225 | (0.204; 0.245) | <0.001 |
| Item8 | 0.092 | (0.038; 0.146) | 0.001 | 0.101 | (0.059; 0.143) | <0.001 |
| Item9 | 0.168 | (0.129; 0.208) | <0.001 | 0.151 | (0.120; 0.182) | <0.001 |
| Item10 | 0.287 | (0.191; 0.382) | <0.001 | 0.260 | (0.182; 0.338) | <0.001 |
| Item11 | -0.019 | (-0.052; 0.015) | 0.273 | 0.030 | (0.004; 0.055) | 0.022 |
| Item12 | 0.268 | (0.231; 0.305) | <0.001 | 0.275 | (0.245; 0.304) | <0.001 |
| Item13 | -1.011 | (-1.067; -0.954) | <0.001 | -1.125 | (-1.171; -1.080) | <0.001 |
| Item14 | -0.115 | (-0.142; -0.087) | <0.001 | -0.094 | (-0.115; -0.073) | <0.001 |
| Item15 | -0.104 | (-0.154; -0.055) | <0.001 | -0.059 | (-0.096; -0.021) | 0.002 |
| Item16 | 0.010 | (-0.036; 0.055) | 0.676 | 0.048 | (0.013; 0.082) | 0.007 |
| Item17 | 0.421 | (0.385; 0.457) | <0.001 | 0.420 | (0.392; 0.449) | <0.001 |
| Item18 | 0.025 | (-0.008; 0.058) | 0.140 | 0.058 | (0.033; 0.083) | <0.001 |
| Item19 | 0.428 | (0.367; 0.490) | <0.001 | 0.493 | (0.444; 0.542) | <0.001 |
| Item20 | 0.439 | (0.408; 0.470) | <0.001 | 0.372 | (0.347; 0.397) | <0.001 |
| Item21 | -0.058 | (-0.094; -0.021) | 0.002 | -0.064 | (-0.092; -0.036) | <0.001 |
| Item22 | 0.253 | (0.207; 0.298) | <0.001 | 0.258 | (0.222; 0.293) | <0.001 |
| Item23 | -0.002 | (-0.029; 0.025) | 0.896 | 0.026 | (0.005; 0.046) | 0.014 |
| Item24 | 0.187 | (0.158; 0.215) | <0.001 | 0.200 | (0.178; 0.222) | <0.001 |
| Item25 | 0.255 | (0.198; 0.311) | <0.001 | 0.186 | (0.139; 0.234) | <0.001 |
| Item26 | 0.007 | (-0.023; 0.038) | 0.631 | -0.010 | (-0.033; 0.013) | 0.395 |
| Item27 | 0.340 | (0.304; 0.377) | <0.001 | 0.366 | (0.337; 0.394) | <0.001 |
| Item28 | 0.381 | (0.352; 0.410) | <0.001 | 0.323 | (0.300; 0.345) | <0.001 |
| Item29 | 0.186 | (0.124; 0.248) | <0.001 | 0.193 | (0.144; 0.242) | <0.001 |
| Item30 | -0.066 | (-0.212; 0.080) | 0.378 | -0.024 | (-0.133; 0.086) | 0.671 |
| Item31 | 0.304 | (0.233; 0.376) | <0.001 | 0.284 | (0.228; 0.339) | <0.001 |
| Item32 | 0.022 | (-0.006; 0.049) | 0.121 | 0.036 | (0.015; 0.057) | 0.001 |
| Item33 | 0.161 | (0.080; 0.241) | <0.001 | 0.190 | (0.126; 0.253) | <0.001 |
| Item34 | 0.153 | (0.121; 0.185) | <0.001 | 0.163 | (0.138; 0.187) | <0.001 |
| Item35 | -0.067 | (-0.131; -0.003) | 0.041 | -0.043 | (-0.095; 0.009) | 0.104 |
| Item36 | 0.319 | (0.289; 0.349) | <0.001 | 0.287 | (0.264; 0.311) | <0.001 |
| Item37 | 0.629 | (0.532; 0.726) | <0.001 | 0.652 | (0.575; 0.730) | <0.001 |
| Item38 | 0.687 | (0.213; 1.161) | 0.005 | 0.831 | (0.490; 1.173) | <0.001 |
| Item39 | - | - | - | - | - | - |
| Item40 | 0.021 | (-0.072; 0.114) | 0.654 | 0.029 | (-0.048; 0.105) | 0.464 |
| Item41 | 1.148 | (1.121; 1.175) | <0.001 | 0.944 | (0.922; 0.966) | <0.001 |
| Item42 | 0.531 | (0.456; 0.606) | <0.001 | 0.547 | (0.489; 0.604) | <0.001 |
