# Supplementary material for: External validation of the Electronic Screening Index of Frailty (e-SIF) in a population of 1.4 million inhabitants aged 65 years and older
Source: Eur J Public Health. 2026 Feb 16;36(2):ckag025. doi: 10.1093/eurpub/ckag025 (PMC13168815; doi:10.1093/eurpub/ckag025)
Supplement: ckag025_Supplementary_Data [file ckag025_supplementary_data.zip › ejph-2025-11-om-0986-File006.docx]

**Supplementary Material. Appendix 1.** One-year mortality and health resource use by frailty category (2018).

|  | **Robust** | **Pre-frail** | **Frail** | **Very frail** | ***P* for trend** |
| --- | --- | --- | --- | --- | --- |
| **Mortality, n (%)** | 13,893 (1.8%) | 18,617 (3.9%) | 14,287 (11.5%) | 9,523 (21.3%) | <0.001 |
| **Hospitalizations, n (%)** | 54,964 (7.1%) | 66,966 (14.1%) | 30,165 (24.3%) | 14,810 (33.1%) | <0.001 |
| Urgent | 38,289 (5.0%) | 50,750 (10.7%) | 26,194 (21.1%) | 13,618 (30.4%) | <0.001 |
| Scheduled | 22,533 (2.9%) | 23,677 (5.0%) | 7,198 (5.8%) | 2,874 (6.4%) | <0.001 |
| Mean (SD) hospitalizations | 0.10 (0.4) | 0.20 (0.6) | 0.37 (0.8) | 0.55 (1.0) | <0.001 |
| Urgent | 0.69 (0.8) | 0.75 (0.9) | 0.89 (1.0) | 1.07 (1.1) | <0.001 |
| Scheduled | 0.60 (0.6) | 0.65 (0.7) | 0.71 (0.8) | 0.82 (0.8) | <0.001 |
| **Institutionalization, n (%)** | 10,189 (1,3%) | 17,726 (3,7%) | 11,657 (9,4%) | 6,818 (15.2%) | <0.001 |
| Mean (SD) institutionalizations | 0.64 (0.8) | 0.69 (0.8) | 0.79 (0.9) | 0.90 (0.9) | <0.001 |
| **Primary care visits, n (%)** | 594,098 (77.1%) | 464,108 (97.5%) | 121,404 (97.7%) | 43,622 (97.5%) | <0.001 |
| Mean (SD) primary care visits | 8.2 (9.7) | 17.9 (15.4) | 25.5 (21.9) | 30.2 (27.7) | <0.001 |
| **Emergency visits, n (%)** | 220,442 (28.6%) | 215,054 (45.2%) | 74,624 (60.1%) | 30,655 (68.5%) | <0.001 |
| Mean (SD) emergency visits | 1.51 (2.2) | 2.05 (2.8) | 3.00 (3.8) | 3.88 (4.9) | <0.001 |
| Median (IQR) emergency visits | 2.35 (2.4) | 2.91 (2.9) | 3.87 (4.0) | 4.69 (5.0) | <0.001 |
| **Day hospital sessions, n (%)** | 10,311 (1.3%) | 12,954 (2.7%) | 4,530 (3.6%) | 1,968 (4.4%) | <0.001 |
| Mean (SD) day hospital sessions | 1.97 (4.3) | 2.03 (4.1) | 2.21 (4.1) | 2.63 (4.4) | 0.638 |
| **Dispensed medications, n (%)** | 453,693 (58.9%) | 419,740 (88.2%) | 112,713 (90.7%) | 41,347 (92.4%) | <0.001 |
| Mean (SD) dispensed medications (€) | 313 (541) | 786 (893) | 1,288 (1,134) | 1,648 (1,279) | <0.001 |

SD: standard deviation. IQR: interquartile range.
